# Polydopamine-Coated Magnetic Nanoplatform for Magnetically Guided Penetration and Enhanced Antibacterial Efficacy in Root Canal Biofilm Elimination

**DOI:** 10.3390/polym17101305

**Published:** 2025-05-10

**Authors:** Xingchen Xu, Pei Wang, Fei Tong, Yifan Liu, Xinyang Hu, Jian Yang, Jun Guo

**Affiliations:** 1 School of Stomatology, Jiangxi Medical College, Nanchang University, Nanchang 330006, China; dusichen94@126.com (X.X.); ndfskqyy620@ncu.edu.cn (P.W.); tongfei015047@126.com (F.T.); lyf070626@163.com (Y.L.); 15798012771@163.com (X.H.); 2 Jiangxi Provincial Key Laboratory of Oral Diseases, Nanchang 330006, China; 3 Jiangxi Provincial Clinical Research Center for Oral Diseases, Nanchang 330006, China

**Keywords:** root canal, magnetic nanoparticles, biofilm, polydopamine, pulpitis

## Abstract

Clinical root canal therapy which takes place through mechanical and chemical strategies is faced with challenges in eliminating bacteria owing to the intricate and curved nature of the root canal system. Moreover, the plaque biofilm within the root canal hinders drug penetration and limits treatment efficacy. Hence, efficient root canal therapy hinges on penetrating into the root canal and overcoming the barriers presented by the plaque biofilms. To penetrate and eradicate biofilms effectively at the root canal, we designed a novel magnetic nanoparticle (MN)-based nanoplatform which was synthesized by the self-polymerization of dopamine on the surface of Fe_3_O_4_ MNs, and then loaded minocycline through the electrostatic interaction. The therapeutic efficacy of minocycline-loaded magnetic nanoparticles (FDM MNs) under a magnetostatic field was observed by various antibacterial experiments. The synthesized FDM MNs exhibited favorable biocompatibility and robust anti-biofilm efficacy. The designed nanoparticles could effectively navigate biofilms to eradicate bacteria residing deep with the assistance of magnetic force. Furthermore, FDM MNs penetrated into dentin tubules under a magnetic field, effectively disrupting biofilms for deep sterilization. The significant results offered valuable experimental evidence to support the potential clinical utility of magnetic nanoparticles for managing pulpitis and periapical inflammation.

## 1. Introduction

Pulpitis and periapical periodontitis are common bacterial infectious oral hard-tissue diseases, which seriously impact the daily life of patients due to the inevitable associated toothache [1]. *Enterococcus faecalis* (*E. faecalis)* is the most commonly detected bacteria in infected root canals [2,3]. Currently, root canal therapy is widely acknowledged as the most effective treatment, removing the inflammatory pulp, necrotic tissue and bacteria in the root canal [4]. However, the efficacy of root canal treatment is currently limited to a success rate of 70–80%, owing to inadequate cleaning of bacteria from the intricate anatomical complexity of the root canal system and the antimicrobial tolerance of bacterial biofilms [5]. On the one hand, it is difficult for instruments to reach the irregular parts (including curved root canals, root isthmus, lateral root canals, C-shaped root canals, etc.), which provide a survival place for bacteria because of large amounts of hard tissue debris, necrotic pulp, and bacterial biofilm [6]. On the other hand, bacterial biofilms exhibit low permeability [7], which impedes drug penetration to confer resistance to antibacterial agents [8,9,10]. Hence, it is imperative to develop a root canal disinfection agent capable of efficiently accessing both the dentin tubule and biofilm to eradicate bacteria. Nanomaterials can transport antibacterial drugs into the root canal system effectively because of their small dimensions, large specific surface area, and superior biochemical properties [11,12,13].

Magnetic nanoparticles (MNs) were utilized to conjugate with various biological functional molecules, including antibacterial drugs, owing to their favorable magnetic attraction, magnetic targeting capabilities, and biocompatibility [14,15,16]. The properties of MNs facilitate the penetration of antibacterial agents into the intricate structure of the root canal curvature and dentin tubules, presenting a novel approach to root canal therapy. Sarah et al. [17] used iron oxide nanoparticles as root canal irrigation to kill *E. faecalis* biofilm in dentin specimens, revealing that MNs penetrated the dentin tubules and biofilm with great bactericidal efficacy. Furthermore, MNs have enhanced the delivery of antimicrobial agents. Quan K et al. [18,19] fabricated gentamicin-loaded MNs to move *Staphylococcus aureus* biofilm via magnetic force. The matrix structure of biofilms could be intensively destroyed by promoting MNs to penetrate bacterial biofilms under the manipulation of magnetic fields. The artificial channels were created by MNs under the magnetic fields to facilitate the penetration of antibiotics, and probably completely remove biofilms [19]. Therefore, the combination of MNs and antibacterial drugs can achieve synergistic antibacterial effects under the manipulation of magnetic force, which is more feasible, innovative, and closer to the clinical conversion for MNs in the field of infection control.

**Figure 1:**
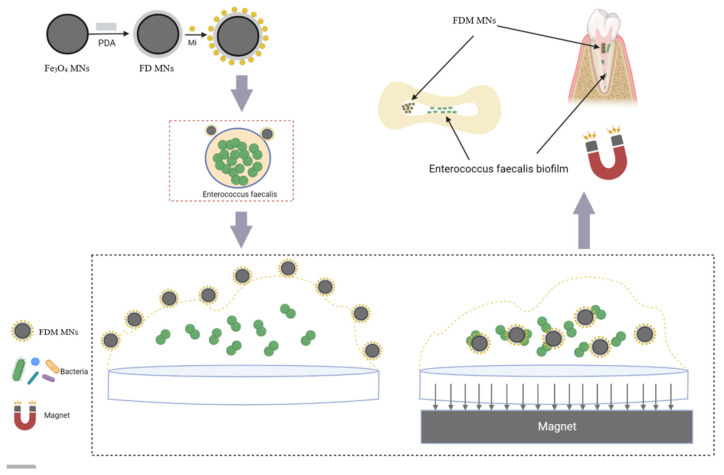
Schematic illustration of preparation of FDM MNs and mechanism for cleaning *E. faecalis* in dentin. Under influence of static magnetic field, FDM MNs demonstrated enhanced directional mobility, facilitating their penetration through root canal space, dentinal tubules, and bacterial biofilm matrices.

Herein, we propose the utilization of a magnetic field to navigate Fe_3_O_4_-based magnetic nanoparticle-loaded minocycline (Mino) (FDM MNs) through the intricate root canal curvature and the bacteria biofilm for further treating pulpitis and periapical inflammation (Figure 1). We hypothesize that magnetically guided delivery of minocycline-loaded nanoparticles (FDM MNs) under a static field enhances dentinal tubule penetration depth compared to passive diffusion, and achieves localized biofilm eradication with reduced antibiotic dosage relative to free drug administration. Initially, ferric oxide nanoparticles undertake facile modification on the surface via the self-polymerization of dopamine in an alkaline solution. Mino, a tetracycline derivative, is widely used to treat Gram-negative and Gram-positive anaerobic bacteria commonly associated with periapical inflammation. Polydopamine (PDA)-coated nanocomposites (FD MNs) contribute to enhancing drug loading through electrostatic interactions and π-π conjugation, resulting in a promotion of antibacterial activity. FDM MNs were utilized to successfully access the dentin tubules and penetrate bacterial biofilms for constructing artificial channels under the magnetic fields, enhancing the antibacterial effect of Mino antibiotics. We underscore the significance of FDM MNs in addressing pulpitis and periapical inflammation when exposed to a magnetic field.

## 2. Materials and Methods

### 2.1. Materials

All the chemical agents were analytical reagents. Iron oxide (II, III) magnetic nanoparticle solution was purchased from Aladdin Biochemical Technology Co., Ltd. (Shanghai, China). Dopamine hydrochloride, Tris (hydroxymethyl) aminomethane, Tris (hydroxymethyl) aminomethane hydrochloride, and minocycline hydrochloride (Mino) were obtained by Aladdin Biochemical Technology Co., Ltd. (Shanghai, China). Brain heart infusion (BHI) broth was provided by Qingdao Hope Bio-Technology Co., Ltd. (Qingdao, China). Hemin was purchased from Hefei BASF Bio-Technology Co., Ltd. (Hefei, China). The Cell Counting Kit-8 (CCK-8) and MTT staining kit were supplied by KeyGen Biotechnology Co., Ltd. (Nanjing, China). The LIVE/DEAD bacterial kit was obtained by Molecular Probes Co., Ltd. (Eugene, OR, USA).

### 2.2. Preparation of FDM MNs

Briefly, 5 mL of Fe_3_O_4_ MNs was dispersed into Tris buffer (pH 8.5) at a concentration of 1 mg/mL, and then 2 mL of 1 mg/mL dopamine hydrochloride solution was added. After stirring for 1 h at room temperature, dopamine self-polymerized on the surface of Fe_3_O_4_ MNs (FD MNs) and FD MNs were collected by magnetic adsorption. To load the antibiotic Mino, 2 mg of FD MNs was added 10 mL of ddH_2_O, and then 2 mL Mino aqueous solution (0.5 mg/mL) in was incorporated with stirring and reacting for 2 h. The Mino-loaded FD MNs (FDM MNs) were obtained by magnetic adsorption and washed twice. Subsequently, the absorbance of supernatant was measured using a UV spectrophotometer (V-750, JASCO, Tokyo, Japan) at 348 nm to calculate the drug loading.

(1) Loading consent (%)=(Mt−Ms)/Mn×100

where Mt is the total weight of Mino; Ms is the total weight of Mino supernatant; Mn is the weight of FDM NPs.

### 2.3. The Characteristics of FDM MNs

For scanning electron microscopy (SEM, JSM-6701F, Nippon, Tokyo, Japan) analysis, freeze-dried nanoparticles (Fe_3_O_4_ MNs, FD MNs, and FDM MNs) were uniformly coated on SEM sample stubs and imaged under high-vacuum conditions. For transmission electron microscopy (TEM, Talos F200X, FEI, Hillsboro, OR, USA), the nanoparticles were dispersed in deionized water, drop-cast onto double-layer copper grids, and air-dried prior to imaging. To determine particle size distribution, the nanoparticles were dispersed in deionized water, and 2 mL of the suspension was transferred to a sample cell for dynamic light scattering (DLS) analysis using a nanoparticle size analyzer (NanoBrook Omni, Brookhaven, Upton, NY, USA).

### 2.4. Cytotoxicity Assay

Human umbilical vein endothelial cells (HUVECs) were obtained from the Shanghai Institute of Cell Medicine, Chinese Academy of Sciences. The cells were cultured in a cell incubator (Thermo Fisher Scientific, Waltham, MA, USA) at 37 °C with 5% carbon dioxide (CO_2_) atmosphere using high-glucose Dulbecco’s Modified Eagle Medium (DMEM, Gibco, Grand Island, NY, USA) containing 10% fetal bovine serum (FBS, PAN Biotech, Aidenbach, Germany), and 1% penicillin-streptomycin (Gibco, Grand Island, NY, USA). HUVECs were seeded in 96-well plates with a density of 5000 cells per well and incubated for 24 h. Fe_3_O_4_ MNs and FD MNs at 0, 20, 50, 100, and 200 μg/mL were added and incubated for 24 h. Therein, the untreated cell was served as a control group. Subsequently, 10 μL of CCK-8 was added to each well. The optical density (OD) was measured at 450 nm using a microplate reader (CMax Plus, Molecular, San Jose, CA, USA), and the relative survival rate of the cells was determined.

### 2.5. The Planktonic Antibacterial Assessment of FDM MNs

#### 2.5.1. Colony-Forming Units (CFUs)

Initially, at a logarithmic phase, *E. faecalis* suspension cultured on brain heart infusion (BHI) medium was diluted to 10^8^ CFU/mL. Then, 0.5 mL *E. faecalis* suspension was added into 0.5 mL BHI medium (as a control group); 0.5 mL Mino solution (20 μg/mL) was dissolved in BHI medium; and 0.5 mL Fe_3_O_4_ MNs, FD MNs, and FDM MNs were dispersed in BHI medium, separately. Subsequently, after co-incubating for 6 h, the suspension was diluted and dispensed onto a BHI agar plate. The plates were transferred to an anaerobic environment and incubated at 37 °C for 24 h. The operation was repeated three times for validation.

#### 2.5.2. Bacterial Growth Curve

The antimicrobial effect of FDM MNs against *E. faecalis* was tested by the time–bacterial growth curve. *E. faecalis* suspension bacteria were diluted to 10^8^ CFU/mL, placed in 48-well plates, and treated with the above-grouped drugs, respectively. After incubation for 0, 1, 2, 4, 6, and 8 h, the 48-well plates were removed to determine the OD value of each well at 600 nm using a microplate reader (Infinite 200 Pro, Tecan, Port Melbourne, Victoria, Austria), and time–bacterial growth curves were plotted.

#### 2.5.3. Metabolic Activity

Briefly, *E. faecalis* suspension was diluted to 10^8^ CFU/mL. The suspension was treated with the above drugs, and 1 mL of MTT staining solution was added to each sample. The samples were placed in an anaerobic box to react at 37 °C for 1 h. Then, the OD value at 600 nm was measured in each well using a microplate reader.

### 2.6. The Anti-Biofilm Assay of FDM MNs

#### 2.6.1. Forming Biofilms in Hydroxyapatite (HA) Disk

HA disks were submerged in saliva for 2 h to facilitate the salivary film formation. Therein, saliva collected from volunteers was centrifuged at 3000 rpm for 20 min to remove cell debris, and the supernatant was filtered through a 0.22 μm bacterial filter. Then, the HA disks were placed at the base of a 24-well plate, where *E. faecalis* was seeded at a concentration of 10^8^ CFU/mL. The culture medium was refreshed daily to form biofilm within approximately four days.

#### 2.6.2. CFU, Metabolic Activity, and Live/Dead Staining

Briefly, 1 mL BHI culture containing different materials was added to a 24-well plate to treat the following biofilms: (i) the control (without any treatment), (ii) Mino, (iii) Fe_3_O_4_ MNs, (iv) FD MNs, (v) FDM MNs, and (vi) FDM MNs + magnetic field, respectively. The concentration of Mino was 50 μg/mL, and that of Fe_3_O_4_ MNs, FD MNs, and FDM MNs was 500 μg/mL with respect to 50 μg/mL Mino. Among them, FDM MNs + magnetic field (200 mT 10 mT, 20 × 10 × 5 mm^3^) means that in the process of treatment, the static magnetic field is applied for 20 min to help the magnetic nanoparticles concentrate on the HA disk and penetrate the biofilm. After 6 h of treatment, HA disks with biofilms were transferred into a new well with 1 mL of BHI broth, and were scraped, sonicated, and vortexed to disperse bacteria. The resulting bacterial suspension was diluted with the culture medium and inoculated onto BHI agar plates. Colonies were enumerated after 24 h of incubation at 37 °C, and CFUs were determined. Each experiment was conducted in triplicate.

Biofilm activity was assessed using an MTT assay. Following rinsing the hydroxyapatite slices with PBS to eliminate unattached bacteria, they were placed in a 24-well plate. Subsequently, 0.5 mL of MTT solution was added to the samples and incubated at 37 °C for 1 h. The resulting blue-violet crystals were dissolved in an equal volume of DMSO. Following agitation for 20 min in a light-protected environment, the DMSO solution was transferred to a 96-well plate, and the OD was measured at 490 nm.

The bacterial live/dead staining kit (Molecular Probes, Eugene, Oregon, USA) comprising NucGreen and EthD-III fluorescent dyes was used to stain HA and dentin slices. NucGreen, a green nucleic acid dye, was used to selectively stain living bacteria, while EthD-III, a red nucleic acid dye, was used to stain dead bacteria specifically. After the above treatments, the staining process followed the protocol in the bacterial live/dead assay kit. Subsequently, the samples were observed using a confocal laser scanning microscope (CLSM, A1 HD25, Nikon, Tokyo, Japan).

### 2.7. Anti-Biofilm Assay in Dentin Disk

Briefly, the no-caries molars were collected from patients who signed the informed consent forms; soft tissues and tartar were removed, and stored in a 4% thymol solution. Then, the collected tooth samples were sliced into dentin slices by a hard tissue microtome (300CP, EXAKT, Berlin, Germany). The enamel and root of the crown were removed by a hard tissue microtome to expose the dentin in the middle of the crown. The dentin pieces were cut into 1.5 × 4 × 4 mm^3^ pieces. The dentin piece specimens were wet-ground with 600-mesh, 800-mesh, 1200-mesh, and 2000-mesh silicon carbide sandpaper, respectively, to remove the secting machine marks and prepare a smear layer with a thickness of 3–5 μm. The dentin slices were ultrasonically wished with sterile water, followed by autoclaving. The surface of dentin slices was acid-etched with 37% phosphoric acid gel for 15 s and thoroughly rinsed with deionized water for 20 s, and the excess moisture on the surface was blown dry to maintain moisture. After acid-etching of tooth samples, the acid-etched dentin surface was thoroughly rinsed with deionized water, the excess moisture on the surface was blown dry, and the surface was kept moist. Under the static magnetic field of the magnet, FDM MN aqueous solution was dripped into the dentin disk for 20 min. Then, the cross section of the mid-region of dentin disks was observed by SEM, with a view as to whether FDM MNs entered the dentin tubule. Subsequently, the dentin slices were incubated with *E. faecalis* at 37 °C for 24 h after being immersed in saliva, just like the HA disk. After four days post-incubation, the bacterial biofilm was grown on the dentin slices. Then, the dentin slices were washed with 1 mL of PBS to remove planktonic *E. faecalis*. The 10 μL of FDM MNs was added to the biofilm. The magnet was positioned at the base for 20 min on the dentin slices to facilitate the magnetic penetration of the FDM MNs into the biofilm. After 6 h of continuous culture, the dentine slices were broken into two pieces and then observed with CLSM.

### 2.8. Statistical Analysis

All data were expressed as the mean ± standard deviation (*n* ≥ 3). The statistical significance of the data was calculated using a one-way analysis of variance (ANOVA) followed by Tukey’s test. A *p*-value of <0.05 was considered statistically significant (* *p* < 0.05, ** *p* < 0.01, *** *p* < 0.001). A *p*-value of >0.05 was considered not significant (ns).

## 3. Results

### 3.1. Characteristics of FDM MNs

**Figure 2:**
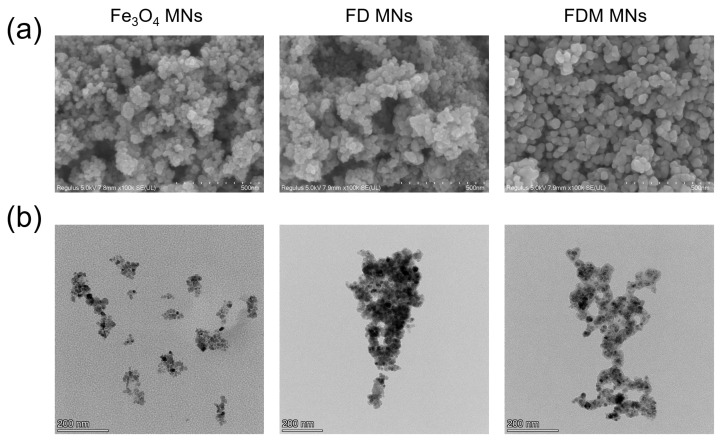
The morphological attributes of the fabricated nanoparticles. (**a**) SEM (scale bar = 500 nm) and (**b**) TEM (scale bar = 200 nm) images of Fe_3_O_4_ MNs, FD MNs, and FDM MNs.

The morphological characteristics of the fabricated nanoparticles were analyzed using SEM (Figure 2a) and TEM (Figure 2b). Microscopic analysis demonstrated that the Fe_3_O_4_ MNs maintained a uniform spherical structure, with an average size of approximately 50 nm. In addition, the results of the DLS analysis (Figure S1) also showed that the average diameters of Fe_3_O_4_ MNs, FD MNs, and FDM MNs were 215 nm, 268 nm, and 275 nm, respectively. The zeta potentials of Fe_3_O_4_ MNs, FD MNs, and FDM MNs were about −28.5, −28.0, and −23.4 mV (Table S1). Surface modification was achieved through in situ self-polymerization of dopamine under alkaline conditions, yielding polydopamine-coated Fe_3_O_4_ MNs (FD MNs). Notably, the polydopamine modification process preserved the spherical morphology of the nanoparticles while inducing a marginal increase in particle size. Subsequent minocycline (Figure S2) loading resulted in the formation of FDM MNs, which exhibited excellent dispersion stability without apparent aggregation, primarily due to the electrostatic stabilization provided by the polydopamine coating. The mapping from TEM revealed Fe enrichment (17.6%) in the nanoparticle core, while N elements (2.3%, shell) uniformly coated the periphery (Figure S3). This dispersion stability represents a critical parameter for biomedical applications, ensuring uniform distribution throughout the root canal system [15]. Moreover, the nanoscale dimensions of FDM MNs (approximately 50 nm) facilitate deep penetration into the intricate root canal anatomy and dentinal tubules when guided by an external magnetic field. The drug release of Mino from FDM MNs at PBS (pH 7.4) is shown in Figure S4, which shows the sustained release of Mino. This innovative approach addresses current limitations in endodontic therapy and represents a significant advancement in the management of intracanal infections.

### 3.2. In Vitro Biocompatibility

**Figure 3:**
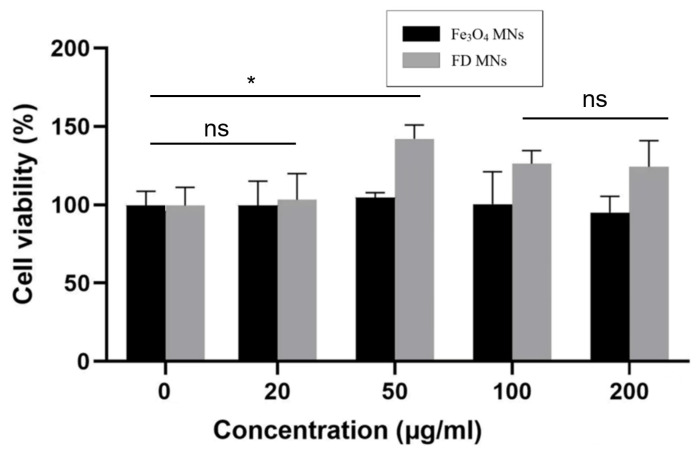
The cellular viability of human umbilical cord cells after culturing with Fe_3_O_4_ MNs and FD MNs for 24 h (ns means not significant. * *p* < 0.05).

Biocompatibility represents a fundamental requirement for biomedical applications of nanoparticles. To evaluate the cytocompatibility of our designed nanocarriers, the CCK-8 method was employed to examine the relative cell viability. Human umbilical cord cells (HUVECs) were co-incubated with Fe_3_O_4_ MNs and FD MNs at various concentrations, with untreated cells serving as the negative control. Quantitative analysis revealed that the cell viability remained above 90% across all tested concentrations (20, 50, 100, and 200 μg/mL) for both Fe_3_O_4_ MNs and FD MNs (Figure 3), as determined by CCK-8 absorbance measurements. These findings indicated that Fe_3_O_4_ MNs and FD MNs demonstrated excellent biocompatibility according to the Biological Evaluation Criteria of Medical Devices (GB/T 16886) [20]. The favorable biocompatibility profile, coupled with maintained cellular viability, underscores the significant potential of these nanocarriers for oral medicine applications.

### 3.3. The Planktonic Antibacterial Capacity of FDM MNs

**Figure 4:**
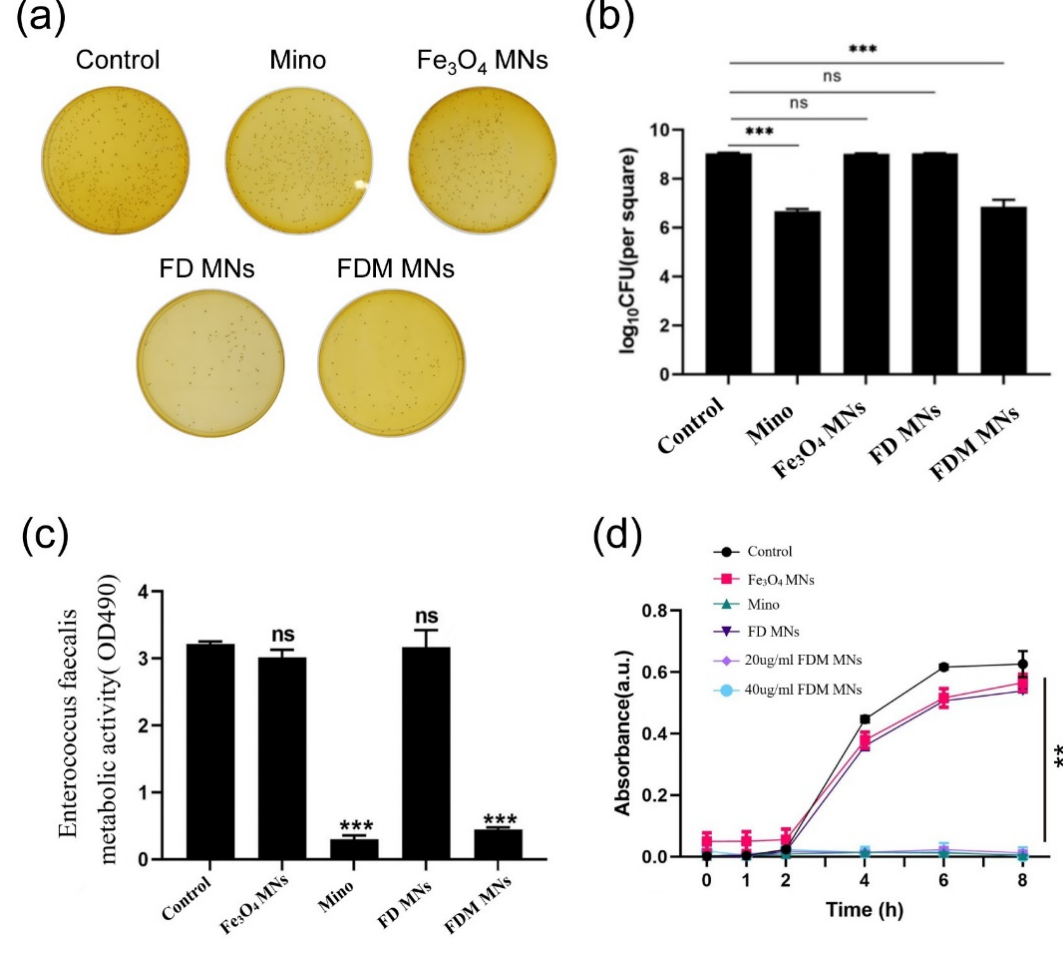
The antibacterial properties of the designed nanoparticles against planktonic bacteria. (**a**) CFU images, (**b**) CFU counts, (**c**) MTT results, and (**d**) the bacterial growth curve of planktonic *E. faecalis* after being treated with Fe_3_O_4_ MNs, Mino, FD MNs, and FDM MNs (ns means not significant. ** *p* < 0.01, *** *p* < 0.001).

To assess the antibacterial efficacy of FDM NPs against planktonic *Enterococcus faecalis*, bacterial CFU counts, bacterial metabolic activity, and time–bacterial growth curves were measured. As displayed in Figure 4a,b and Figure S5, the results showed that CFU counts of Fe_3_O_4_ MNs and FD MNs groups did not obviously differ from the control group. In contrast, both minocycline (Mino) and FDM MNs groups exhibited significantly reduced bacterial counts (*p* < 0.05), indicating potent bactericidal activity. Furthermore, parallel measurements of bacterial metabolic activity corroborated these findings (Figure 4c). The metabolic profiles of the Fe_3_O_4_ MN and FD MN groups showed no significant difference from the control group, whereas the Mino and FDM MN groups displayed markedly reduced metabolic activity. Moreover, the time-dependent growth kinetics analysis for FDM NPs against *E. faecalis* suspension was shown in Figure 4d. The antibacterial efficacy of Fe_3_O_4_ MNs and FD MNs against *E. faecalis* suspension bacteria was comparable to that of the control group, suggesting the absence of intrinsic antimicrobial properties in these formulations. Notably, FDM MNs demonstrated comparable initial bactericidal efficacy to free minocycline within the first hour of exposure. However, the nanoparticle formulation exhibited sustained antimicrobial activity, maintaining prolonged inhibitory effects on planktonic *E. faecalis* populations. This extended antimicrobial profile highlights the advantage of the nanocarrier system over conventional antibiotic administration.

### 3.4. The Anti-Biofilm Capacity of FDM MNs

**Figure 5:**
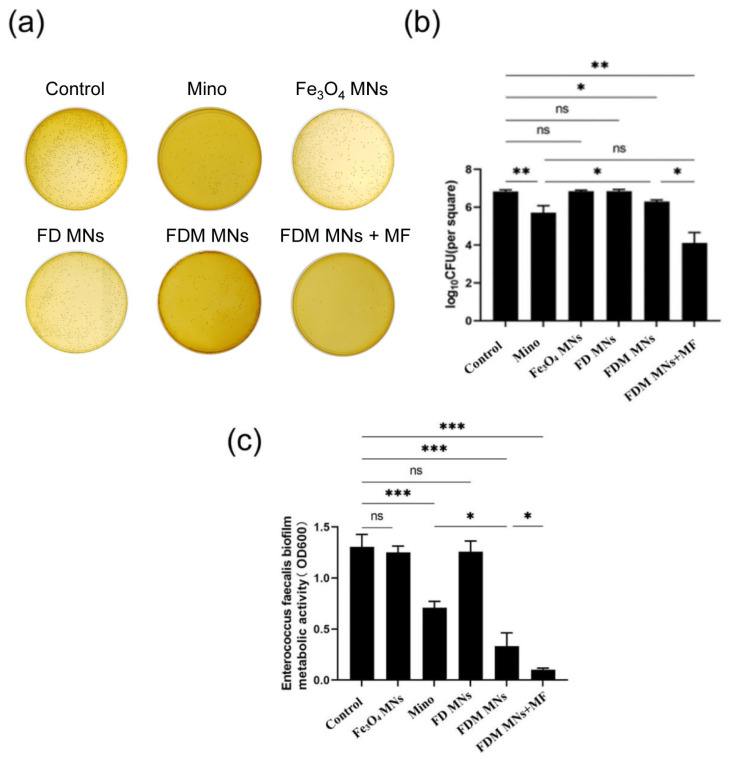
The anti-biofilm property of the designed nanoparticles. (**a**) CFU images, (**b**) CFU counts, and (**c**) MTT results of *E. faecalis* biofilms after being treated with FDM MNs under the magnetostatic field (ns means not significant. * *p* < 0.05, ** *p* < 0.01, *** *p* < 0.001).

To investigate the anti-biofilm efficacy of FDM MNs under magnetostatic field guidance, we established an experimental system incorporating permanent magnets to generate controlled magnetic fields for nanoparticle targeting. Biofilm formation was induced on hydroxyapatite (HA) disks to simulate oral cavity conditions, followed by quantitative assessment of antimicrobial efficacy through CFU enumeration and metabolic activity analysis using MTT assay. To ensure the maturity of the biofilm, the crystal violet staining of biofilm was measured (Figure S6). As shown in Figure 5a,b and Figure S7, quantitative analysis revealed distinct *E. faecalis* biofilm inhibition patterns among treatment groups. While Fe_3_O_4_ MNs and FD MNs showed comparable CFU counts to the untreated control, FDM MNs without magnetic field application demonstrated significant biofilm reduction (*p* < 0.05). Notably, both minocycline solution and magnetically guided FDM MNs groups exhibited substantially lower CFU counts compared to the control group. Moreover, The CFU counts of magnetically guided FDM MNs groups are the lowest after treatment. The metabolic activity profile of *E. faecalis* biofilms (Figure 5c) closely correlated with CFU enumeration results. Neither Fe_3_O_4_ MNs nor FD MNs significantly affected biofilm metabolic activity compared to the control group. In contrast, FDM MNs without application of the magnetic field demonstrated moderate metabolic inhibition, while both the minocycline solution and magnetically guided FDM MN groups exhibited substantially reduced metabolic activity. Importantly, the enhanced anti-biofilm efficacy of magnetically guided FDM MNs was statistically equivalent to the free minocycline solution (*p* > 0.05), while significantly surpassing the performance of FDM MNs without application of the magnetic field (*p* < 0.05). These findings demonstrate that magnetic field guidance significantly enhances the anti-biofilm efficacy of FDM MNs, achieving comparable performance to conventional antibiotic treatment while maintaining the advantages of targeted drug delivery.

**Figure 6:**
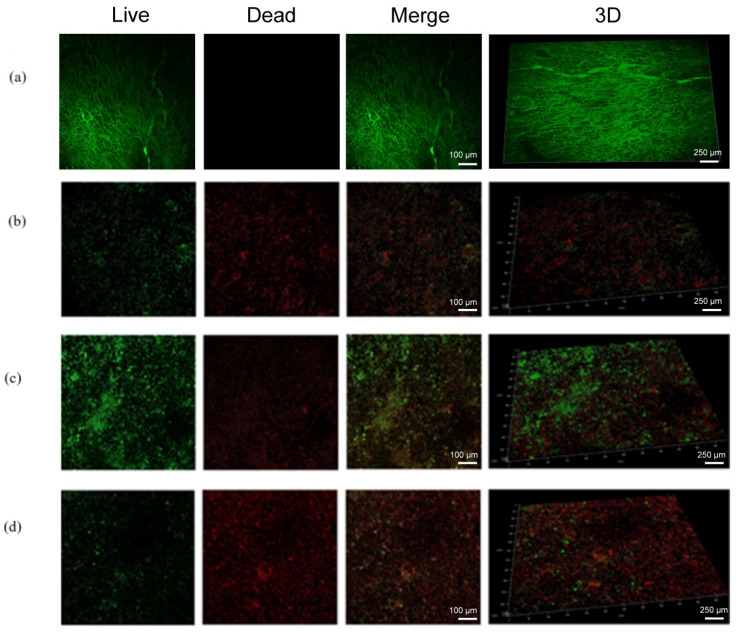
The CLSM fluorescent images of *E. faecalis* biofilms stained with a live/dead staining kit on HA disks after treatments: (**a**) control, (**b**) Mino, (**c**) FDM MNs, (**d**) FDM MNs + MF.

To further characterize the anti-biofilm activity of FDM MNs on HA surfaces, we performed CLSM analysis using a live/dead bacterial viability kit. This dual-staining approach enabled simultaneous visualization of viable (green fluorescence) and non-viable (red fluorescence) bacterial populations, with overlapping regions appearing yellow or orange, indicating mixed populations within the biofilm architecture. Representative CLSM images (Figure 6) revealed distinct patterns of biofilm viability across treatment groups. Untreated control biofilms exhibited dense green fluorescence, indicating robust viability of *E. faecalis* (Figure 6a). Comparative analysis of treatment groups included both low-magnification panoramic views and high-resolution images, with separate channels showing red and green fluorescence distributions (Figure 6b–d). The results demonstrated significant differences in biofilm viability between treatment modalities. While both minocycline solution and FDM MNs without the magnetic field application showed moderate antimicrobial activity, the magnetically guided FDM MN group exhibited markedly enhanced efficacy. This was evidenced by predominant red fluorescence (indicating non-viable bacteria) with minimal residual green fluorescence in the magnetically targeted group, suggesting near-complete biofilm eradication. The spatial distribution analysis revealed that magnetic field guidance significantly improved nanoparticle penetration and antimicrobial activity throughout the biofilm structure. These findings provide visual evidence supporting the superior anti-biofilm efficacy of magnetically guided FDM MNs, demonstrating their potential for effective biofilm eradication in endodontic applications.

### 3.5. Anti-Biofilm Capacity of FDM MNs in Dentin Disk

**Figure 7:**
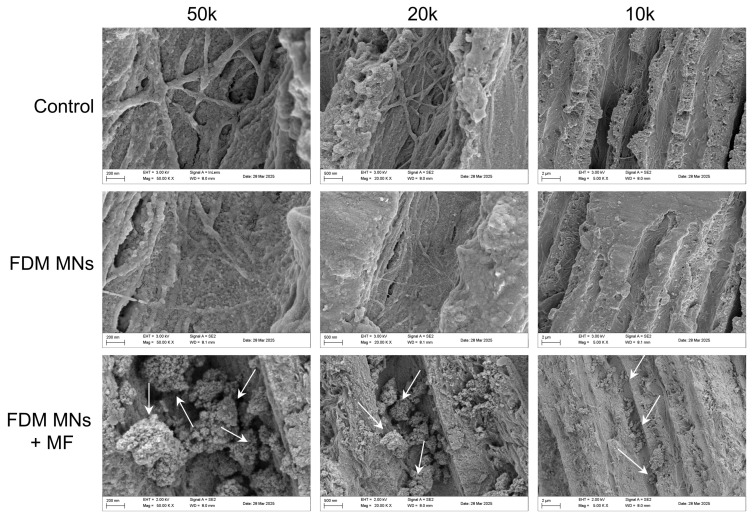
SEM images used to observe FDM MNs penetrating the dentin tubules under the attraction of a magnetic field. The SEM images show dentin tubules with different magnifications. Magnetic nanoparticles in dentin tubules can be observed in the FDM MNs + MF group, but not in the other two groups. The arrows point to FDM MNs in the dentinal tubules.

To investigate whether FDM MNs could penetrate dentinal tubules under the guidance of a static magnetic field, dentin slices were fractured and analyzed via SEM to visualize the nanoparticle distribution within the dentin tubules. As shown in Figure 7, FDM MNs successfully entered and aggregated within dentin tubules under the MF application, as observed in the mid-region of the dentin disk, demonstrating that nanoparticles of this size can be magnetically guided into the tubular structure. In contrast, without MF, no obvious FDM MNs were observed in tubules, likely due to rapid fluid runoff from the dentin surface carrying away the nanoparticles before penetration. After calculation, the proportion of FDM MNs in dentinal tubules is approximately 75%. Moreover, energy-dispersive spectroscopy (EDS) was further employed to map the atomic distribution and quantify elemental composition (C, O, P, Ca, Fe) across experimental groups (Figure S8 and Table S2). It revealed that the FDM MNs + MF group exhibited a significantly higher Fe content (~4 atomic%), confirming the successful magnetic-driven accumulation of nanoparticles within dentinal tubules. These results collectively validate the efficacy of static magnetic fields in directing FDM MNs into dentinal microchannels, overcoming challenges posed by fluid dynamics in non-magnetic conditions.

**Figure 8:**
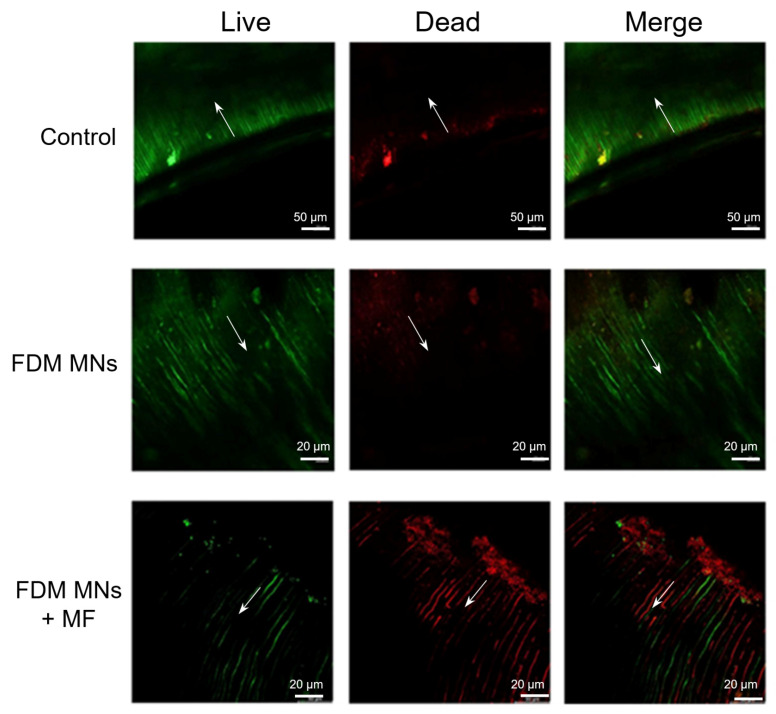
The CLSM fluorescent images of *E. faecalis* biofilms stained with a live/dead staining kit on dentin disks after treatments. The direction of the arrow is the direction of the dentin tubule. The green fluorescence is the living bacteria, and the red fluorescence is the dead bacteria.

To further evaluate the antibacterial capacity of FDM MNs for dentin, live/dead stained images of the *E. faecalis* biofilm within dentin slices were analyzed by CLSM. In the absence of magnetic field application, CLSM images (Figure 8) revealed a distinct spatial distribution pattern: while the biofilm surface exhibited predominant red fluorescence (indicating bacterial death), dentinal tubules displayed persistent green fluorescence (suggesting viable bacterial populations). It can be seen from the control group in the figure that the depth of the bacterial biofilm in the dentinal tubules is 100–200 μm, which may be related to the growth time of the bacteria. The observation indicates that FDM MNs effectively eliminate surface-adherent bacteria but demonstrate limited penetration through the biofilm matrix into dentinal tubules without magnetic guidance. Notably, magnetic field application significantly enhanced the antimicrobial performance of FDM MNs (Figure 8 and Figure S9), which revealed increased red fluorescence intensity within dentinal tubules. This enhanced penetration suggests that magnetic field guidance facilitates nanoparticle transport through the biofilm matrix and into dentinal structures. However, the limited penetration depth indicates that while magnetic targeting improves antimicrobial efficacy, complete eradication of deeply entrenched bacteria within dentinal tubules remains challenging. These results demonstrate that magnetic field application significantly enhances the penetration capability and antimicrobial efficacy of FDM MNs in dentin, although complete eradication of intratubular bacteria may require optimization of nanoparticle properties or magnetic field parameters.

## 4. Discussion

Current clinical practice predominantly relies on sodium hypochlorite (NaOCl) and chlorhexidine for root canal irrigation; however, these conventional agents are limited by poor dentin permeability and transient antimicrobial effects. Inspired by the directional movement and aggregation of magnetic nanoparticles under a magnetic field, we synthesized FDM MNs to address some issues, such as passing through the dentin tubule and the long-term reservation of antibacterial agents. Nanomaterials have become prevalent in the field of dentistry due to their size and multi-functionality, such as in endodontics, periodontics, oral and maxillofacial surgery, and orthodontics [21]. While enzymatic/redox-active nanocarriers show promise in simple biofilms, their reliance on specific biochemical cues limits efficacy in polymicrobial root canal infections [19]. Our magnetic propulsion strategy is biofilm-agnostic, enabling universal penetration regardless of extracellular matrix composition. Existing studies have demonstrated a classical strategy wherein superparamagnetic nanoparticles are surface-immobilized on biomaterial substrates. Following biofilm colonization on these functionalized surfaces, external magnetic fields are employed to extract nanoparticles, thereby creating perfusion channels through the biofilm matrix. This microarchitecture modification significantly enhances antibiotic penetration for efficacious microbial eradication [18]. Divergent from this established paradigm, our study pioneers a magnetically guided delivery system. Through precisely controlled magnetic gradients, we achieve directional transport of antibiotic-loaded iron oxide nanoparticles into dentinal tubules and through biofilm barriers, facilitating targeted release of nanoparticle-encapsulated vancomycin within biofilm microenvironments. Herein, our designed FDM MNs could penetrate biofilms and effectively eradicate bacteria, thereby addressing the challenges associated with the limited efficacy of antimicrobial agents. FDM MNs were synthesized via the self-polymerization of dopamine to create a multifunctional platform incorporating Fe_3_O_4_ MNs and loaded with Mino. These nanoparticles exhibited uniform morphology, with a particle size of approximately 50 nm, significantly smaller than the minimum diameter of dentin tubules (500 nm) [22,23]. Smaller nanoparticles have potential to penetrate deeper into root canals, enhancing disinfection by effectively reaching and navigating narrow, curved areas more easily [24]. However, the inherent limitations of passive diffusion often restrict nanoparticle penetration depth within dentinal structures. To overcome this barrier, we implemented magnetic field guidance, which provides controlled directional force to enhance nanoparticle transport. This approach represents a significant advancement over conventional nanoparticle delivery systems, potentially enabling more effective treatment of deep dentin infections that are typically inaccessible to traditional antimicrobial agents. While ex vivo experiments showed that FDM MNs penetrated dentinal tubules under static fields, the clinical translation to curved molar canals (e.g., S-shaped mesiobuccal canals) requires optimization. In addition, although short-term assays showed >90% HUVEC viability, we added recommendations for long-term hepatic/renal monitoring in future animal studies.

Notably, FDM MNs effectively inhibited the growth of an *E. faecalis* suspension for an extended duration. In addition to conventional disinfection (antibiotics, NaOCl, and chlorhexidine), a number of new antimicrobial methods have also been used in root canals [25,26,27]. The treatments have shown promise in root canal treatment through their potent bactericidal effects and ability to combat drug-resistant pathogens; they face challenges in biofilm penetration. Complementing these biochemical approaches, physical methods like photothermal therapy and magnetic field manipulation have emerged as effective biofilm disruption strategies. Recent advancements in nanotechnology have introduced novel biofilm eradication strategies, including enzymatic EPS degradation and redox-active nanoparticles. Specifically, nanoplatforms incorporating dextranase have demonstrated the ability to cleave polysaccharide chains, thereby destabilizing biofilm architecture [28,29]. Additionally, redox-active nanoparticles, such as silver nanoparticles, disrupt biofilm integrity through interactions with sulfur-containing amino acids in EPS proteins, altering their tertiary structure and function [30]. Complementing these biochemical approaches, physical methods like photothermal therapy and magnetic field manipulation have emerged as effective biofilm disruption strategies [31,32]. Our findings demonstrate that magnetic field guidance significantly enhances the antimicrobial efficacy of FDM MNs, facilitating both biofilm penetration and intracellular antimicrobial activity. While direct comparison with NaOCl will be pursued in future clinical simulations, our system’s key advantage lies in targeted antibiotic delivery. Free minocycline was selected as the primary control to isolate magnetic guidance’s contributions. Quantitative analysis revealed a logarithmic reduction in *E. faecalis* biofilm viability, as evidenced by CFU enumeration and MTT assay results. Confocal microscopy analysis of live/dead stained biofilms confirmed the superior antimicrobial performance of magnetically guided FDM MNs, demonstrating effective biofilm eradication on dentin surfaces and penetration into dentinal tubules. These results establish the combination of FDM MNs with magnetic field stimulation as a promising therapeutic strategy for combating persistent *E. faecalis* biofilm infections in endodontic treatment. However, there has been no further exploration on the depth at which magnetic nanoparticles enter dentinal tubules. We are conducting research and will publish it in future articles.

## 5. Conclusions

We synthesized multifunctional FDM MNs by co-encapsulating dopamine hydrochloride and Mino onto the surface of Fe_3_O_4_ nanoparticles. The FDM nanoparticles exhibited favorable characteristics such as good dispersity, chemical stability, and biocompatibility. Additionally, our study presents a novel magnetic field-guided strategy to overcome the anatomical challenges of root canal systems by utilizing FDM MNs. This approach capitalizes on the synergistic combination of magnetic targeting and controlled antibiotic delivery, enabling effective penetration into complex root canal anatomies, dentinal tubules, and biofilm matrices. The magnetic guidance system significantly enhanced minocycline delivery efficiency, achieving dual therapeutic outcomes: (1) superior biofilm eradication within the root canal system, and (2) targeted disinfection of dentinal tubules through nanoparticle penetration. While this proof-of-concept study demonstrates promising results, several limitations warrant consideration. First, the long-term biocompatibility of residual Fe_3_O_4_ nanoparticles requires thorough investigation, as prolonged iron ion release may potentially induce cytotoxicity in periapical tissues. Second, the current formulation lacks controlled drug release mechanisms, raising concerns about potential antibiotic leakage and associated allergic reactions. Third, precise spatial control of magnetic fields needs optimization to ensure consistent nanoparticle navigation through intricate root canal morphologies. These technical challenges highlight critical areas for future research: (1) the development of biodegradable magnetic carriers, (2) the integration of stimulus-responsive drug release systems, and (3) the implementation of real-time magnetic field modulation technologies.

## Data Availability

Data are contained within the article and Supplementary Materials.

## Supplementary Materials

The following supporting information can be downloaded at https://www.mdpi.com/article/10.3390/polym17101305/s1, Figure S1: Representative images showing the size distribution of Fe_3_O_4_ MNs, FD MNs, and FDM MNs by DLS; Figure S2. The structure of Minocycline. Figure S3. Elemental mapping based on TEM image presenting location of Fe, O, and N elements in FDM MNs; Figure S4. The drug release of Mino from FDM MNs at PBS (pH 7.4); Figure S5: CFU images of planktonic *E. faecalis* after being treated with Fe_3_O_4_ MNs, Mino, FD MNs, and FDM MNs; Figure S6. The crystal violet staining of biofilm before treatment; Figure S7: CFU images of *E. faecalis* biofilms after being treated with FDM MNs under the magnetostatic field; Figure S8: Elemental mapping based on SEM image presenting the location of C, O, P, Ca, and Fe elements on dentin disks after treatments; Figure S9. The bacteria death rate from the quantification CLSM live/dead fluorescence intensity; Table S1: The energy dispersive spectroscopy (EDS) analysis of various elements; Table S2. The energy dispersive spectroscopy (EDS) analysis of various elements; Video S1: A video of the designed magnetic nanoparticles without static-magnetic field. Video S2: A video of static-magnetic-field attraction for the designed magnetic nanoparticles.

## Abbreviations

CFU	Colony-forming unit
ESD	Energy dispersive spectroscopy
EPS	Extracellular polymers
FD MNs	Dopamine on the surface of Fe_3_O_4_ magnetic nanoparticles
FDM MNs	Minocycline-loaded magnetic nanoparticles
MNs	Magnetic nanoparticles
Mino	Minocycline
OD	Optical density
PDA	Polydopamine
