# Supplementary material for: Polydopamine-Coated Magnetic Nanoplatform for Magnetically Guided Penetration and Enhanced Antibacterial Efficacy in Root Canal Biofilm Elimination
Source: Polymers (Basel). 2025 May 10;17(10):1305. doi: 10.3390/polym17101305 (PMC12115043; doi:10.3390/polym17101305)
Supplement: Supplementary file 1 [file polymers-17-01305-s001.zip › polymers-3609288-supplementary.docx]

**Novel Fe_3_O_4_ magnetic nanomaterials loaded with minocycline killing *Enterococcus Faecalis* biofilm in root canal infection *in vitro***

Xingchen Xu^a, b, c#^, Pei Wang^a, b, c#^, Fei Tong^a, b, c^, Yifan Liu^a, b, c^, Xinyang Hu^a, b, c^, Jian Yang^a, b, c*^ Jun Guo^a, b, c*^

a. School of Stomatology, Jiangxi Medical College, Nanchang University, Nanchang 330006, PR China

b. Jiangxi Provincial Key Laboratory of Oral Diseases, Nanchang 330006, PR China

c. Jiangxi Provincial Clinical Research Center for Oral Diseases, Nanchang 330006, PR China

^#^ These authors contributed equally to this work.

* Corresponding authors: ([jianyang@ncu.edu.cn](mailto:jianyang@ncu.edu.cn), J. Yang), ndfskqyy320@ncu.edu.cn (J. Guo).


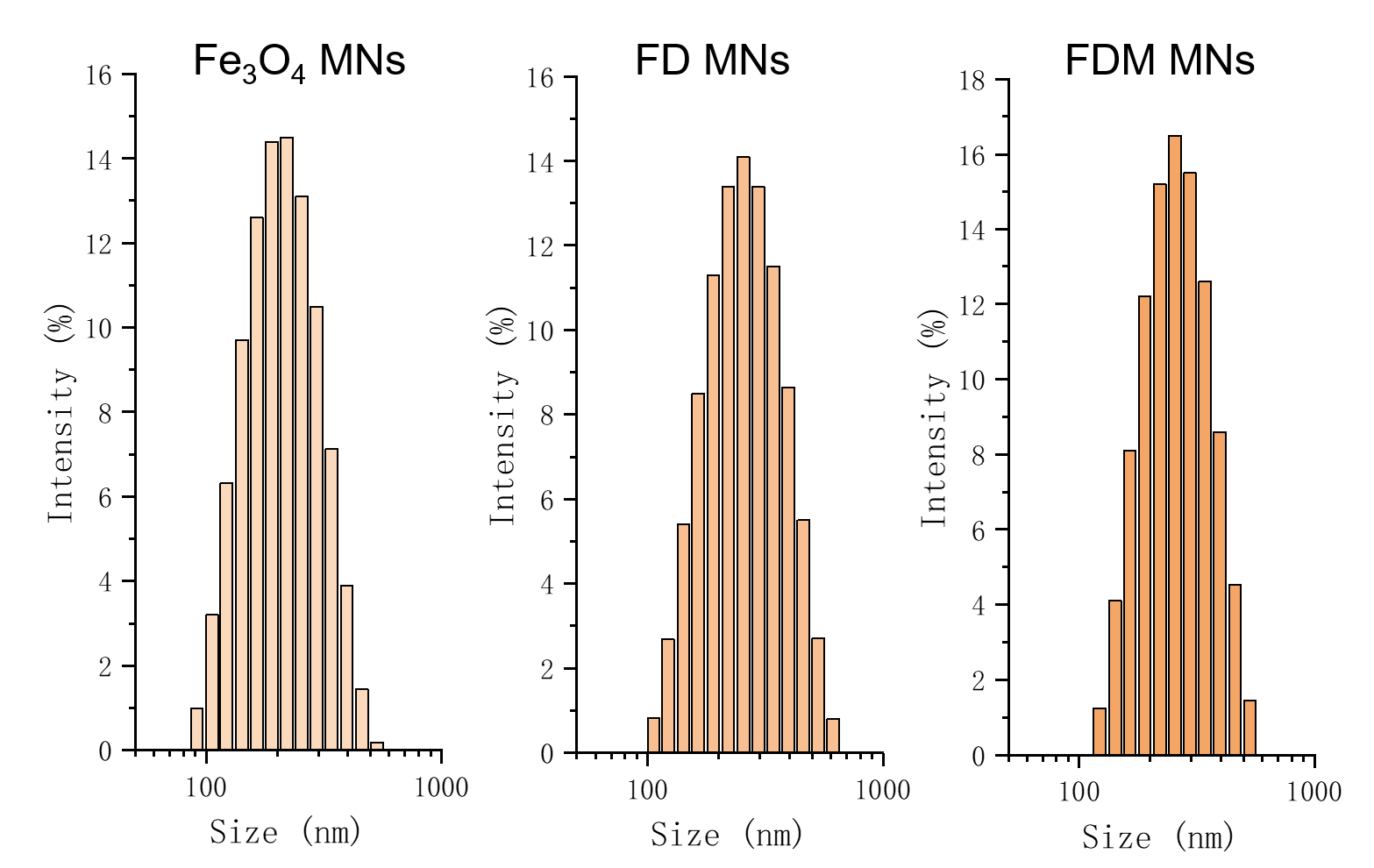


**Figure S1.** Representative images showing the size distribution of Fe_3_O_4_ MNs, FD MNs and FDM MNs by DLS.


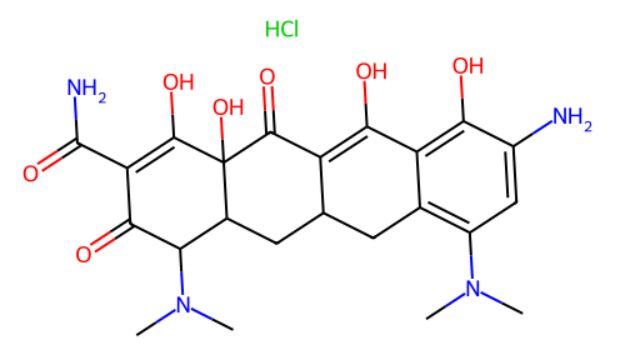


**Figure S2**. The structure of Minocycline.


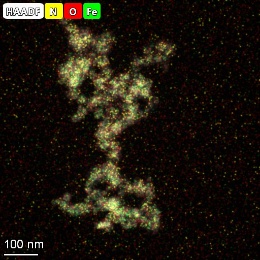

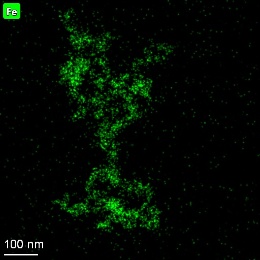

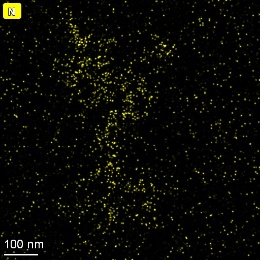

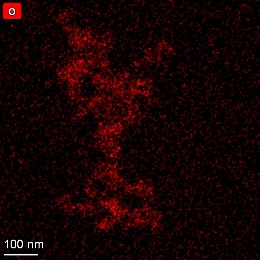


**Figure S3.** Elemental mapping based on TEM image presenting the location of Fe, O, and N elements in FDM MNs.


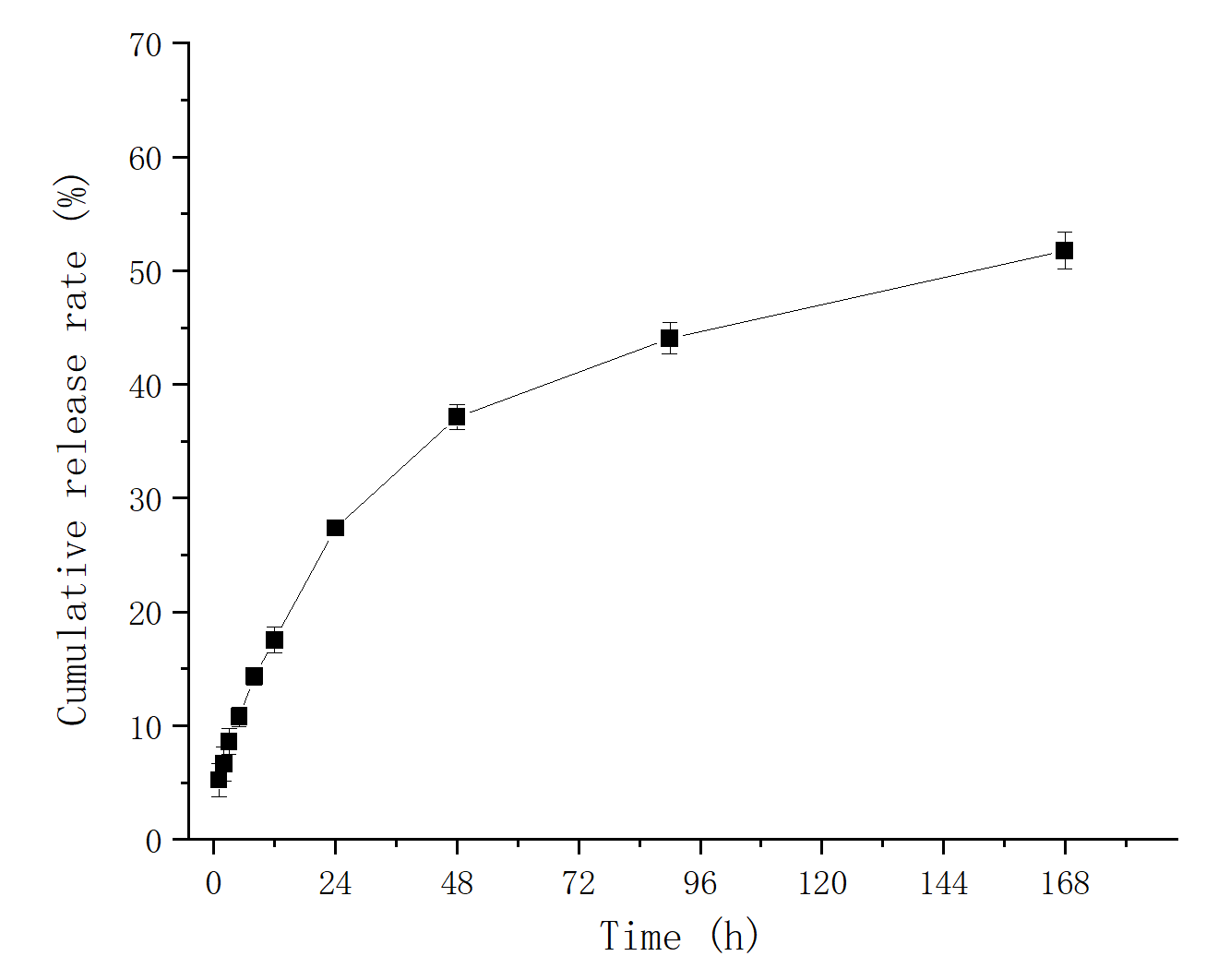


**Figure S4.** The drug release of Mino from FDM MNs at PBS (pH 7.4).


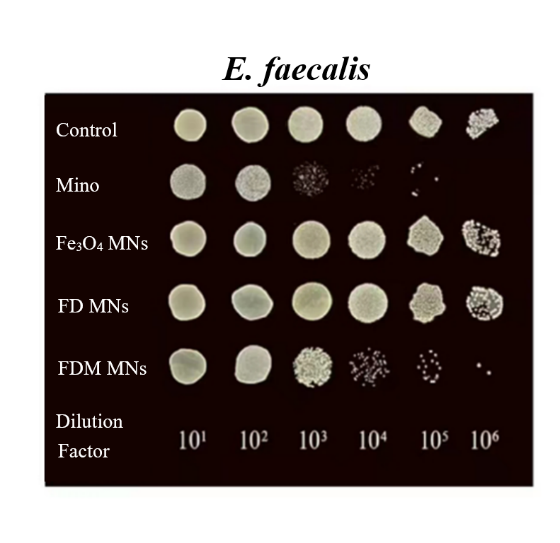


**Figure S5.** CFU images of planktonic *E. faecalis* after being treated with Fe3O4 MNs, Mino, FD MNs and FDM MNs.


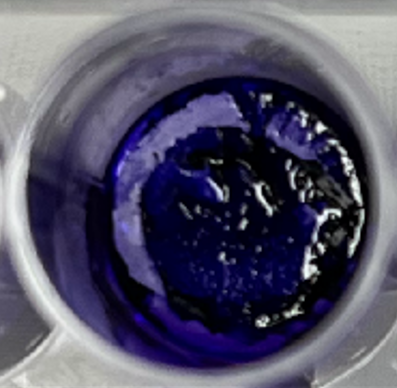


**Figure S6.** The crystal violet staining of biofilm before treatment.


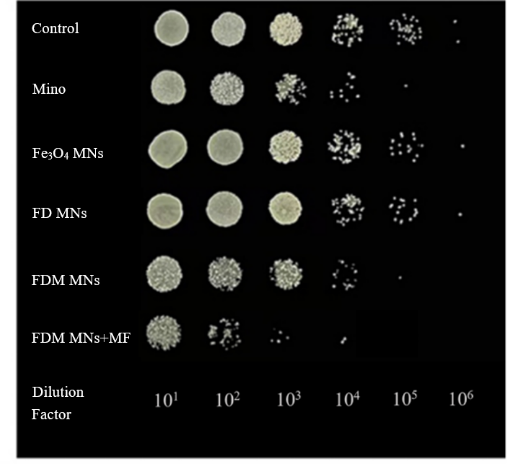


**Figure S7.** CFU images of *E. faecalis* biofilms after being treated with FDM MNs under the magnetostatic field.


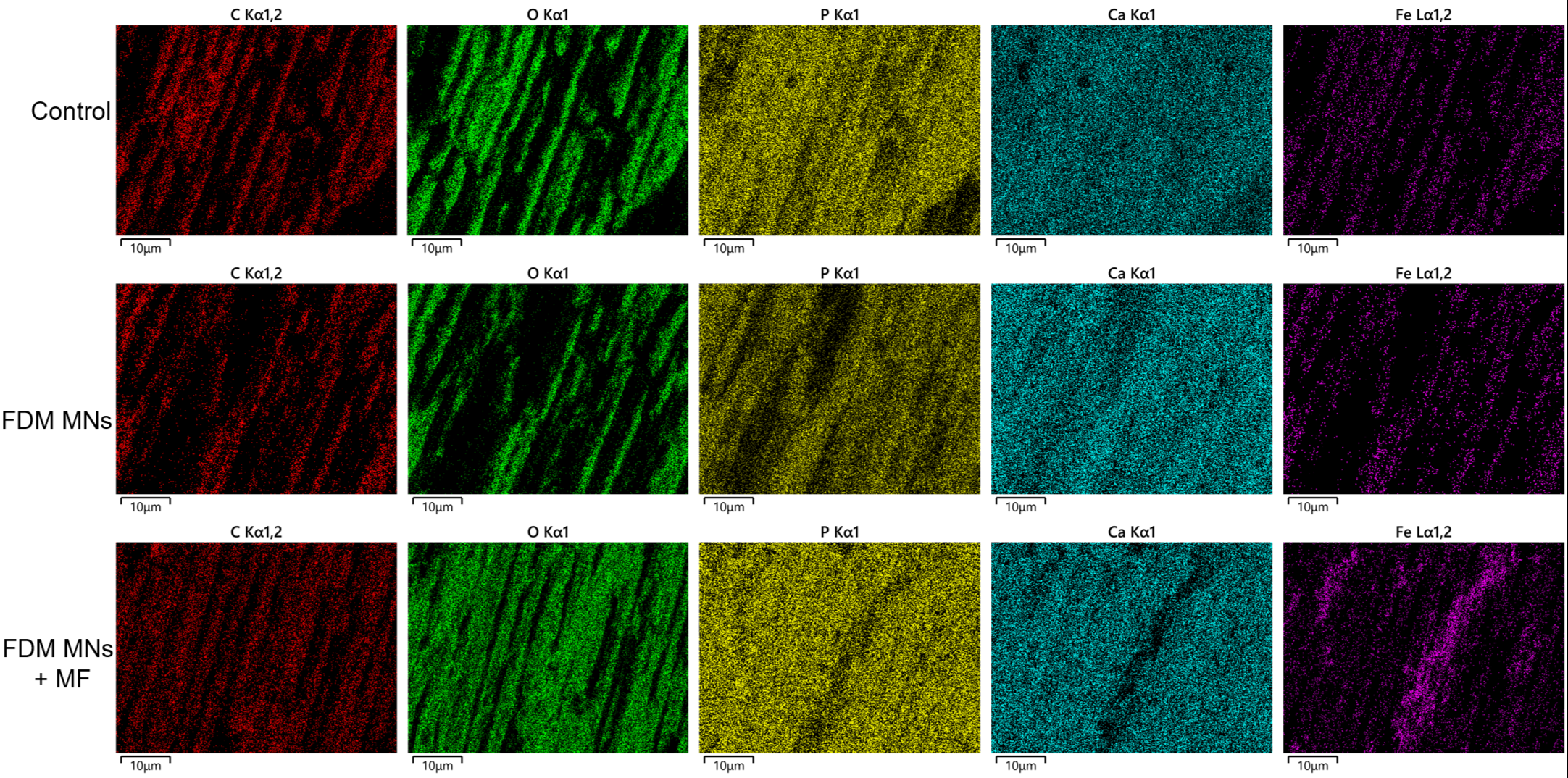


**Figure S8.** Elemental mapping based on SEM image presenting the location of C, O, P, Ca and Fe elements on dentin disks after treatments.


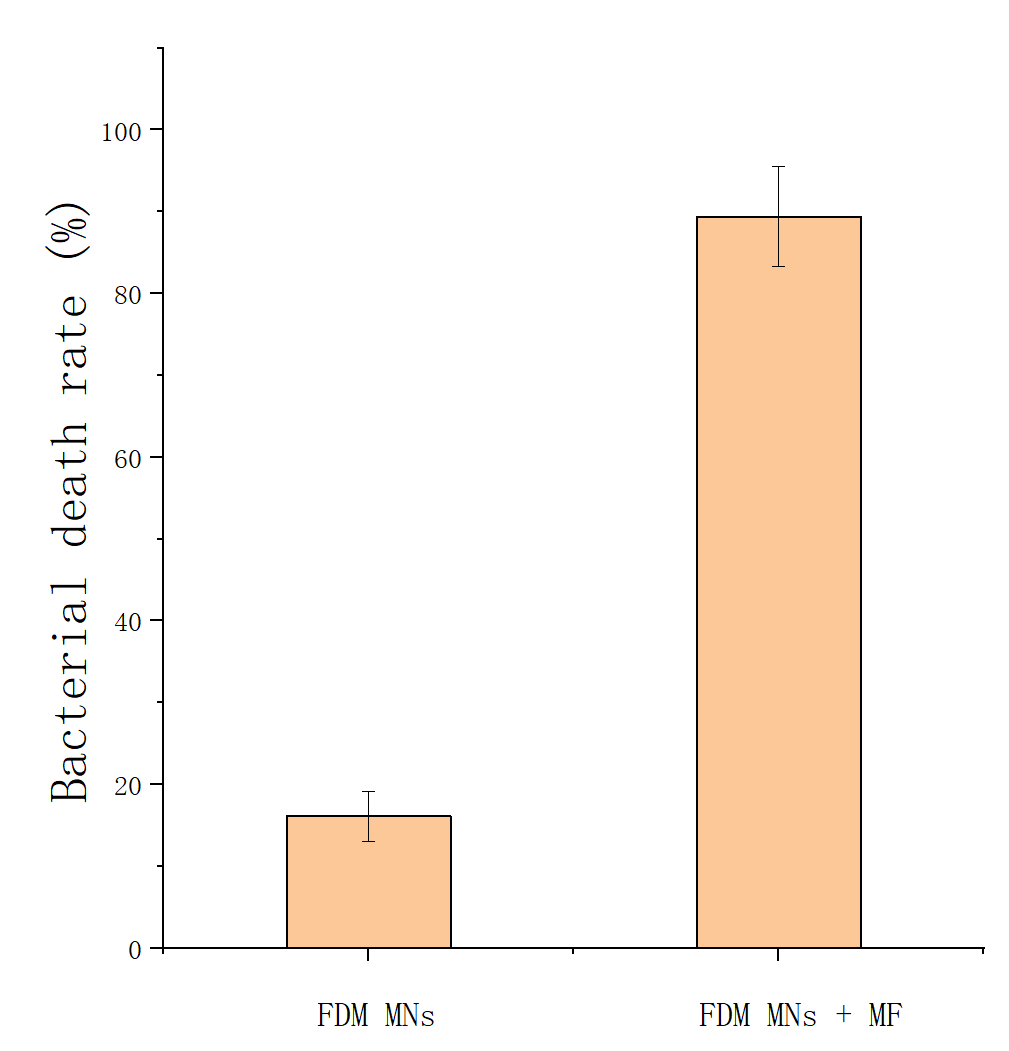


**Figure S9.** The bacteria death rate from the quantification CLSM live/dead fluorescence intensity.

**Table S1.** The zeta potential of three nanoparticles.

| Samples | Fe_3_O_4_ MNs | FD MNs | FDM MNs |
| --- | --- | --- | --- |
| Zeta potential | -28.5 ± 1.5mV | -28.0 ± 0.9 mV | -23.4 ± 1.3 mV |

**Table S2.** The energy dispersive spectroscopy (EDS) analysis of various elements.

| Groups | Atomic percent | | | | |
| --- | --- | --- | --- | --- | --- |
|  | C | O | P | Ca | Fe |
| Control | 32.22% | 43.68% | 8.38% | 14.79% | 0.93% |
| FDM MNs | 22.76% | 43.98% | 10.31% | 21.68% | 1.28% |
| FDM MNs + MF | 26.80% | 51.45% | 6.80% | 10.87% | 4.08% |
